# Sex-Related Differences of Acute and Chronic Pancreatitis in Adults

**DOI:** 10.3390/jcm10020300

**Published:** 2021-01-15

**Authors:** Madeline Drake, Shah-Jahan M. Dodwad, Joy Davis, Lillian S. Kao, Yanna Cao, Tien C. Ko

**Affiliations:** Department of Surgery, UT Health Houston, Houston, TX 77030, USA; Madeline.M.Drake@uth.tmc.edu (M.D.); Shah-Jahan.Dodwad@uth.tmc.edu (S.-J.M.D.); Joy.M.Davis@uth.tmc.edu (J.D.); Lillian.S.Kao@uth.tmc.edu (L.S.K.)

**Keywords:** acute pancreatitis, chronic pancreatitis, sex-associated differences, epidemiology, animal models

## Abstract

The incidence of acute and chronic pancreatitis is increasing in the United States. Rates of acute pancreatitis (AP) are similar in both sexes, but chronic pancreatitis (CP) is more common in males. When stratified by etiology, women have higher rates of gallstone AP, while men have higher rates of alcohol- and tobacco-related AP and CP, hypercalcemic AP, hypertriglyceridemic AP, malignancy-related AP, and type 1 autoimmune pancreatitis (AIP). No significant sex-related differences have been reported in medication-induced AP or type 2 AIP. Whether post-endoscopic retrograde cholangiopancreatography pancreatitis is sex-associated remains controversial. Animal models have demonstrated sex-related differences in the rates of induction and severity of AP, CP, and AIP. Animal and human studies have suggested that a combination of risk factor profiles, as well as genes, may be responsible for the observed differences. More investigation into the sex-related differences of AP and CP is desired in order to improve clinical management by developing effective prevention strategies, diagnostics, and therapeutics.

## 1. Introduction

Acute pancreatitis (AP) is a painful and sudden inflammatory condition of the pancreas and is the most common gastrointestinal diagnosis requiring hospitalization [1]. The incidence of AP is increasing in the United States, with a 25.2% increase in AP-related hospital admissions from 2001 to 2014 [1,2,3]. Though originally thought to be two distinct diseases, it has been shown that recurrent episodes of AP can lead to the development of chronic pancreatitis (CP), a condition characterized by progressive inflammation and fibrosis. It is now suggested that the two conditions exist on a spectrum, emphasizing the importance of understanding their etiologies and pathogeneses [4,5].

It is well known that a combination of risk factors and genetics contribute to the development of pancreatitis; however, the impact of sex is not well understood. Investigation into whether sex is associated with the development, progression or outcomes of pancreatitis is warranted. Thus, this current literature review provides an updated investigation and foundation for future studies regarding the sex-related differences in AP and CP.

## 2. Sex Differences in Pancreatitis

AP is the most common disease to affect the pancreas and the most common diagnosis upon hospital admission for gastroenterological disease [5]. Numerous etiologies of AP and CP have been described, as shown in Table 1, while the impacts of sex are still actively being investigated [5,6]. In 2016, there were nearly 520,000 AP and CP hospitalizations in the United States, with 48% being female [7,8]. Although the incidence of AP is similar among males and females, male sex is associated with higher mortality [9]. Additionally, men are more likely to develop recurrent acute pancreatitis (RAP), which can lead to the development of CP by healing areas of necrosis with fibrotic tissue. Thus, men also develop CP at higher rates compared to women (12 cases per 100,000 versus 6 cases per 100,000, respectively) [4,5,10,11]. The most common etiologies of AP are gallstone pancreatitis and alcohol-induced pancreatitis, while the single most common etiology of CP is alcohol-induced [6]. However, the incidence of etiology differs when stratified by sex, as shown in Table 1. Therefore, we discuss the most common etiologies of AP and CP adjusted by sex in the following sections.

### 2.1. Gallstone Pancreatitis

Gallstone pancreatitis is the most common etiology of AP and accounts for 24.7% of cases in the United States and 35.0% of cases worldwide [2,12,13,14,15,16,17,18,19,26]. The prevalence of gallstone pancreatitis increases with age, with the highest rates in both sexes seen in ages 75+, consistent with the epidemiology of gallstones in the general population [6]. Furthermore, the incidence of gallstone pancreatitis has been increasing. It is hypothesized to be secondary to increasing rates of obesity, a primary risk factor for the development of gallstones [6]. Krishna et al. found that compared to 2002–2005, in 2009–2012 there was a more than 3-fold increase in metabolic syndrome and morbid obesity, which was associated with a 13.2% increase in AP hospital admissions [1].

Reported rates of gallstone pancreatitis are significantly higher among women, representing up to 30.2% of cases compared to 19.3% of cases in men for all-cause AP [2,12,26,27]. Although it is a more common etiology in women, specifically among gallstone pancreatitis, more adverse outcomes are associated with male sex. After risk-adjustment, odds of complications related to gallstone pancreatitis and death are higher in men [28].

### 2.2. Alcohol-Induced

Alcohol-induced AP is the second most common cause of AP, representing 25.2% of cases in the US and 20.4% of cases worldwide [2,12,13,14,15,16,17,18,19,26,29]. Although a single episode of alcohol consumption can induce alcohol-related AP, chronic consumption is a major risk factor for the development of pancreatitis, with as much as a 4-fold increase in prevalence among subjects with a history of alcoholism [30]. Alcohol-related pancreatitis is also the most common etiology of CP, responsible for up to 49.0% of cases [6,31].

Alcohol-induced AP is more common in men, with males representing 72.8% of cases in the United States [2,29,32]. Men are also more likely to have alcohol-related CP, while non-alcoholic etiologies account for up to approximately 70.0% of CP cases in women [4,6,10,20,21,33]. Age of onset varies by sex in alcohol-related CP, with women having a peak incidence from age 35–44 and men from age 45–54 [34].

Notably, the sex differences for alcohol-induced AP and CP are diminished when similar levels of alcohol consumption are compared, thus suggesting the difference is likely due to higher rates of alcohol consumption in men [6,31,35]. However, recent studies suggest that genetic factors may also play a role. For example, variants in the X-linked gene, CLDN2, may modulate the risk for alcohol-induced pancreatitis and therefore partially explain the higher rates of alcohol-induced pancreatitis among men [36,37].

### 2.3. Metabolic

#### 2.3.1. Hypertriglyceridemia

Hypertriglyceridemia (HTG) is a well-established cause of AP, accounting for up to 10.4% of cases worldwide [14,38]. HTG-induced AP typically develops in patients with an underlying genetic abnormality, such as in familial combined hyperlipidemia or familial HTG, usually in the presence of a secondary factor such as alcohol use or uncontrolled diabetes. Importantly, HTG-induced AP is associated with a severe clinical course, and patients commonly have recurrent attacks leading to frequent hospitalizations and an increased risk of CP [39].

Rates of HTG-induced AP are higher in men than in women, with men representing 67.3% of cases [2,12,14,22,26,40]. The increased rates in men are likely due to higher rates of secondary factors, such as concurrent alcohol use and metabolic comorbidities [14]. Of note, pregnancy, hormone replacement therapy, and the use of oral contraceptive pills are risk factors for HTG-induced AP, as estrogens increase triglyceride levels by simulating very low density lipoprotein production in the liver [39,41]. In one study of pregnant women with AP, HTG was the etiology in 30.0% of cases [38].

#### 2.3.2. Hypercalcemia

Hypercalcemia is a well-known risk factor for pancreatitis when total ionized calcium levels are ≥12.0 mg/dL or 3 mmol/L (typical upper limit of normal = 10.2 mg/dL). Over 90.0% of cases of hypercalcemia are due to primary hyperparathyroidism (PHPT) or hypercalcemia of malignancy, though less than 7.0% of patients with PHPT develop AP [42].

Interestingly, women are affected by PHPT two to three times as often as men; however, rates of AP secondary to PHPT are significantly higher among men (6.9%) compared to women (2.2%) [23,43]. This may be due to compounding risk factors, such as increased rates of alcohol intake or metabolic conditions in men, as previously described. Further investigation is needed to see if there is a sex-related link to AP secondary to PHPT.

### 2.4. Trauma

Post-traumatic pancreatitis is rare in the United States, representing between 0.1 and 3.5% of cases worldwide [10,12,18,20]. Two common types of trauma leading to pancreatitis are blunt abdominal trauma and post-endoscopic retrograde cholangiography. Overall, post-traumatic pancreatitis is more common among men than women, with men representing 63.6% of cases in the United States [10,20]. However, the disease mechanisms regarding the relationship between trauma and chronic pancreatitis are not clear.

#### 2.4.1. Abdominal Trauma

Pancreatitis or pancreatic injury following abdominal trauma (both penetrating and blunt) is rare in adults [44,45,46]. The most common mechanism is blunt abdominal trauma, involving rapid force against the pancreas, resulting in possible rupture secondary to compression against the spinal vertebrae [44,47]. Siboni et al. completed a retrospective review of the National Trauma Data Bank and found that pancreatic injury had an overall incidence of 3.0%, with most injuries being low-grade [45]. Isolated pancreatic injury occurred in <1% of all abdominal injuries or 20.9% of all pancreatic injuries. Of the isolated blunt pancreatic injuries, 63.0% occurred in males [45]. Most cases are managed non-operatively [47]. In a separate retrospective review of the National Trauma Data Bank, Kuza et al. reviewed all trauma patients age >14 and found the incidence of pancreatic trauma to be 0.3%. Nearly 75.0% of these patients were male [44].

Given the rare nature of this injury, there are limited data available given to specifically identify gender differences in pancreatic injury or pancreatitis following abdominal trauma. Since there is an overall higher incidence of trauma in males, it would not be unreasonable to assume that there is a higher incidence of pancreatitis or pancreatic injury following abdominal injury in males. However, further investigation is needed to review clinical outcomes of these patients adjusted by sex.

#### 2.4.2. Post-Endoscopic Retrograde Cholangiopancreatography (ERCP) Pancreatitis

Pancreatitis is the most common complication of post-ERCP. The incidence of post-ERCP pancreatitis varies among studies due to patient factors, procedures, and methodology; however, most studies demonstrate an incidence between 3.0 and 5.0% [48,49].

It is commonly thought that young women are at higher risk of post-ERCP pancreatitis, though this association is controversial. Several studies have found female sex to be an independent risk factor for the development of post-ERCP pancreatitis [18,50,51]. In 2015, a meta-analysis of 28 studies demonstrated an odds ratio (OR) of 1.46 for females [52]. Still, others have found no association [53,54,55]. It has been suggested that other risk factors may increase the risk of post-ERCP pancreatitis synergistically. For instance, Sphincter of Oddi dysfunction, an independent risk factor for the development of post-ERCP pancreatitis, is more common in women overall, which could make the actual association between women and post-ERCP pancreatitis difficult to distinguish [56]. Furthermore, Freeman et al. found the highest risk of post-ERCP pancreatitis (42%) in female patients with normal serum bilirubin, Sphincter of Oddi dysfunction, and difficult biliary cannulation [50].

### 2.5. Autoimmune

Autoimmune pancreatitis (AIP) is a rare form of CP that accounts for fewer than 2.4% of cases in the United States and is classified into two subtypes [6]. Type 1 AIP, or lymphoplasmacytic sclerosing pancreatitis, is characterized by IgG4 positive plasma cells and lymphocytes [57,58]. Type 2 AIP, or idiopathic duct-centric pancreatitis, is IgG4 negative.

In contrast to most autoimmune diseases that preferentially affect young women, the overall male to female ratio of AIP is 2.94:1 [24]. The most affected age group also differs between males and females (70–79 versus 60–69 years of age) [24]. Furthermore, changes to sex preferences are observed when adjusted for subtypes of AIP. For instance, no sex differences have been observed in Type 2 AIP [58]. In contrast, men older than 60 years of age are more likely to have Type 1 AIP [58]. Type 1 AIP is generally thought to be related to IgG4-related systemic diseases with extrapancreatic manifestations, such as primary sclerosing cholangitis, an autoimmune destruction of the bile ducts, also more commonly seen in males [57].

### 2.6. Other

#### 2.6.1. Smoking

Smoking is an independent risk factor for both AP and CP [6]. Several studies have demonstrated that smoking can lead to pancreatic acinar cell injury through the elevation of intracellular calcium levels and/or the impairment of pancreatic blood flow [59]. Continued smoking worsens the progression of CP, and smokers have a 2-fold increased risk of pancreatic cancer compared to non-smokers [59,60,61].

Tobacco use is greater in men than in women, and is highly associated with alcohol consumption [35]. Pancreatic calcifications in CP are more common in men than in women and are associated with heavy smoking (≥20 cigarettes/day) [35]. Interestingly, disease counseling of CP typically focuses on alcohol cessation, while smoking cessation is clinically underemphasized despite being an independent risk factor [59].

#### 2.6.2. Malignancy

Pancreatic cancer is responsible for approximately 3.0% of all cancers in the United States. The incidence of pancreatic cancer between 2013 and 2017 was 26.5 per 100,000, with 56.1% of cases occurring in men [62]. The higher rates of pancreatic cancer in men are thought in part to be due to higher rates of smoking, as discussed above. Mortality rates from pancreatic cancer are also greater among men (12.6 per 100,000) compared to women (9.6 per 100,000) [63].

Pancreatic duct obstruction secondary to malignancy may lead to AP and RAP. Due to the relatively rare nature of this condition, there are few population-based studies and, thus, the true epidemiology is not well known. In one study investigating intraductal papillary mucinous neoplasm (IPMN), a precancerous neoplasm of the pancreas, 6.9% of patients were found to have AP/RAP secondary to IPMN [64]. Though 85.0% of patients with IPMN were male, rates of secondary AP/RAP were similar among both sexes. In another study investigating patients with AP or RAP secondary to periampullary tumors, two-thirds of the patients were male [25]. However, the results of the study are limited secondary to small sample size (*n* = 15).

#### 2.6.3. Medication-Induced

Many medications have been proposed to induce AP, but none are known to cause CP. Drug-induced AP (DIAP) is responsible for less than 3.0% of AP cases. However, the actual incidence may be higher due to underreporting and challenges associated with the diagnosis, such as in patients with multiple comorbidities and underlying risk factors for AP [65]. Incidence also varies among medication, with some known pancreatotoxic drugs, such as azathioprine and didanosine, having incidences as high as 5.0% and 23.0%, respectively [65].

While it is difficult to ascertain whether medication use impacts pancreatitis development by sex, certain medications used more commonly by one sex may demonstrate associations. An example of this includes estrogen-containing products, which increase the risk of HTG-induced AP, as described above [39]. Otherwise, sex was not associated with an increased risk of DIAP secondary to the use of other medication classes known to cause DIAP, such as thiopurines, highly active antiretroviral therapy, angiotensin-converting enzyme inhibitors, or valproic acid [66,67,68,69].

#### 2.6.4. Pregnancy

Pregnancy, being unique to the female population, has rarely been associated with AP, occurring in approximately 3 out of every 10,000 pregnancies [70]. A majority of these cases are diagnosed in the third trimester or in the postpartum period [70,71]. Prior to the utilization of ERCP, AP was associated with 20% and 50% mortality rates in mothers and fetuses, respectively [70]. However, with ERCP, mortality rates have significantly decreased, approaching <2.0% mortality in mothers and <5.0% risk of fetal loss [70,72].

The most common cause of AP in pregnant patients is gallstone pancreatitis, accounting for almost 70% of cases, followed by hypertriglyceridemia [70,73]. By itself, gallstone pancreatitis or acute cholecystitis complicating pregnancy occurs in approximately 0.05–0.8% of all pregnancies. There is increasing risk of gallstone pancreatitis with increasing number of pregnancies. Therefore, if non-operative strategies are used to manage a patient in the third trimester, it is recommended that patients undergo cholecystectomy as soon as safely possible [73,74].

The increased incidence of gallstone pancreatitis in the third trimester is secondary to two physiologic changes. Firstly, there is an increased secretion of cholesterol relative to bile acid and phospholipids in bile. This causes a “super-saturation” of bile, leading to the precipitation of stones. Secondly, the rate at which the gallbladder empties is decreased, while the volume of bile stored in fasting and postprandial states is increased. This combination leads to the favorable formation of gallstones [70].

HTG-induced AP can also occur in pregnancy. A 3-fold rise in serum triglyceride levels in the third trimester has been reported secondary to the increased estrogen-induced synthesis of triglycerides [70]. As previously mentioned, HTG is a known etiology of AP.

Rarer causes of AP in pregnancy include hyperemesis in the first trimester, hyperparathyroidism, preeclampsia, and acute fatty liver of pregnancy [70].

## 3. Clinical Presentation and Management

The most common clinical symptom of both AP and CP is abdominal pain. AP can present on a spectrum of severity, from mild abdominal pain to severe organ failure with abdominal compartment syndrome requiring surgical intervention. The management of AP is based on the primary etiology. There is limited literature on clinical outcomes of AP adjusted by sex, which would require further investigation. CP symptoms include abdominal pain, vitamin deficiencies, steatorrhea, and other signs of malabsorption. Pain experience, morphology and malabsorption in CP are not significantly different between men and women; however, males are more likely to develop the comorbidity of diabetes mellitus [10]. Rates of CP-related disability and medical therapies used are similar in both sexes; however, sphincterotomy is performed more frequently in women. In contrast, cyst/pseudocyst procedures are more common in men [33]. Detailed information and guidance regarding the management of AP and CP are reviewed in other publications [75,76,77].

## 4. Animal Studies

### 4.1. Animal Models of Pancreatitis

As essential translational studies, animal models of pancreatitis have been designed to mimic human pancreatitis in order to further examine the mechanisms of pathogenesis [78]. Although animal studies on pancreatitis typically use male animals, the animal studies using both males and females, or with sex hormone treatment, also suggest that sex may play a role in the development of AP and CP. For example, in a mouse model of AP, mice were fed a choline-deficient diet enriched with ethionine, a known pancreatotoxin, and severe AP with hemorrhagic necrosis was induced in female mice, but not in males. However, when male mice were treated with estradiol, severe AP developed at similar rates as females fed the same diet. Treatment with estradiol alone had no effect on the pancreas, suggesting that the hormone may potentiate the toxicity of ethionine [79]. This finding is consistent with the association of AP in humans with estrogen-containing medications, such as oral contraceptive pills and hormone replacement therapy [39].

In another model, mice with genetically impaired autophagy developed CP similar to that of humans, but in a sex-dependent manner. Female mice developed CP at a lesser rate compared to male mice, consistent with human epidemiological studies. Female mice also had less reactive oxygen species (ROS) accumulation, and had preserved exocrine and endocrine pancreatic tissue [80]. Taken together, the results suggest an ROS-dependent, sex-specific effect of autophagy. This finding is consistent with other studies that have shown ROS detoxification is affected by androgen and estrogen receptor activity, thus the ability to recover from ROS-mediated tissue damage is sex-dependent [81].

Murphy Roths Large Lymphoproliferative (MRL/Mp or MRL/lpr) mice are commonly used to study autoimmune disease, while a modified version of MRL/Mp mice, MRL/+, which do not contain the lymphoproliferative gene, lpr, will develop the same diseases but with lessened severity and at an increased age of onset. One study using the MRL/+ mice found that AIP developed in a sex-dependent manner. Male mice were less likely to develop the disease, but if present, the AIP was more likely to develop later in life with a lessened severity compared to female mice [82].

Interestingly, the MRL/Mp mouse model of AIP also involved multiple organs and elevations in serum auto-antibodies. This most closely resembles the IgG4 positive Type 1 AIP, which in humans is more common in males [57,83]. However, though more clinically similar to Type 1 AIP, the MRL/Mp mouse model of AIP is IgG4 negative [83]. In this model, sex hormones are known to impact the titers of autoantibodies, and treating female mice with androgens has been shown to slow the progression of autoimmune disease [82].

However, not all animal models of pancreatitis have demonstrated sex-dependent associations. For instance, using the cerulein-induced mouse CP model, our research group assessed gross and histological injury and recovery following cerulein injection. Both male and female mice showed similar responses to the pancreatic injury induced by cerulein injection for 4 weeks and recovered partially 4 weeks later after cessation of the cerulein injection [84]. Similarly, in the L-arginine-induced mouse model of AP, no significant differences in the severity of disease between males and females were observed [85].

### 4.2. Suggested Mechanisms

Several pathways have been suggested to modulate the sex-associated differences observed in animal models. One study using corticotropin-releasing hormone (or factor) receptor 2-deficient *(Crhr2^−/−^)* mice suggests that sex-specific stress-responses are involved. Kubat et al. showed that urocortin 1 (Ucn1), an anti-inflammatory mediator, was expressed by pancreatic acinar cells of wild type (WT) males at baseline, while not expressed by WT females. In cerulein-induced AP, *Crhr2^−/−^* male mice showed attenuated de novo Ucn1 induction, as well as increased inflammation and necrosis. While *Crhr2^−/−^* females showed similar levels of injury, treatment with exogenous Ucn1 reduced histological damage and cellular stress response in males only. Thus, the Crhr2 and Ucn1 mediated-stress response may be protective in male but not in female mice [86]. However, further investigation is needed to review clinical outcomes in humans adjusted by sex.

Furthermore, pancreatic acinar cells are known to contain estrogen receptors, suggesting that estrogen could play a role in the development or progression of pancreatic disease. Exogenous estrogen has been shown to accumulate in the pancreas, and when administered to rats and dogs, has been shown to increase the weight of the pancreas [79].

Nuclear estrogen receptors (ER) -α, -β, and the transmembrane G protein-coupled estrogen receptor (GPER) have been implicated in many gastroenterological diseases, but the effects are less characterized in the pancreas. For instance, one study showed that estradiol treatment in hepatic stellate cells suppressed fibrogenesis via ERβ in a dose-dependent manner [87]. In another study, a mouse model of hepatocellular carcinoma was used to show that GPER knockout in a diethylnitrosamine-induced tumor accelerated tumorigenesis, immune cell infiltration, and fibrogenesis [88]. However, the role of GPER in the inflammation and fibrogenesis of AP and CP has yet to be studied.

## 5. Conclusions

Sex-dependent associations are seen in human and animal AP and CP; however, the underlying mechanisms are unclear. Steroid hormone-related stress response and direct effects of sex hormones, such as estrogen, may play a role. Regarding the novelty and clinical significance, sex-dependent responses to diseases including pancreatitis are important and increasing clinical problems. In our opinion, this review focuses on a literature review of sex-dependent responses to pancreatitis from reported studies in the United States and worldwide, which should be informative for clinical management and translational studies. More research is needed to elucidate the sex-dependent pathomechanistic differences of AP and CP, so that more effective clinical management strategies in prevention, diagnostics, and therapeutics may be developed.

## Figures and Tables

**Table 1:** Common Etiologies Respective to Acute or Chronic Pancreatitis (AP/CP) by Sex.

Etiology	%AP (%Male)	Country [Reference]	CP (%Male)	Country [Reference]
Gallstone	24.7 (39.3)	USA [2]	3.0 (72.7)	Japan [10]
	25.8 (51.5)	Japan [12]		
	40.0 (37.0)	Australia [13]		
	55.8 (53.7)	China [14]		
	36.9 (39.6)	UK [15]		
	31.8 (42.2)	Germany [16]		
	23.5 (39.6)	Ireland [17]		
	49.9 (39.2)	Norway [18]		
	26.8 (40.3)	Sweden [19]		
Average	35.0 (42.5)		3.0 (72.7)	
Alcohol	25.2 (72.8)	USA [2]	44.5 (70.0)	USA [20]
	32.6 (87.9)	Japan [12]	69.7 (92.3)	Japan [10]
	22.0 (79.0)	Australia [13]	71.6 (81.7)	Germany [21]
	10.0 (97.2)	China [14]		
	22.0 (77.7)	UK [15]		
	34.5 (87.8)	Germany [16]		
	23.2 (75.3)	Ireland [17]		
	9.7 (83.6)	Norway [18]		
	4.8 (80.5)	Sweden [19]		
Average	20.4 (82.4)		61.9 (81.3)	
Hypertriglyceridemia	4.9 (65.6)	USA [2]	6.1 (51.5)	USA [20]
	2.3 (69.7)	Japan [12]	0.9 (50.0)	Japan [10]
	10.4 (72.9)	China [14]		
	2.3 (61.1)	UK [22]		
Average	5.0 (67.3)		3.5 (50.8)	
Hypercalcemia	NA * (78.0)	France [23]	0.6 (66.7)	USA [20]
			0.6 (71.4)	Japan [10]
Average	NA * (78.0)		0.6 (69.1)	
Trauma	0.1 (62.5)	Japan [12]	2.0 (63.6)	USA [20]
	3.5 (20.0)	Norway [18]	0.3 (66.7)	Japan [10]
Average	1.8 (41.3)		1.2 (65.2)	
Autoimmune	NA *	NA *	2.4 (46.2)	USA [20]
			NA * (75)	Japan [24]
Average			2.4 (60.6)	
Malignancy	3.6 (66.7)	China [25]	0.4 (0)	USA [20]
Average	3.6 (66.7)		0.4 (0)	

* NA = Not available.
